# Detection of HPV infection in urothelial carcinoma using RNAscope: Clinicopathological characterization

**DOI:** 10.1002/cam4.4091

**Published:** 2021-06-23

**Authors:** Fidele Y. Musangile, Ibu Matsuzaki, Mitsuaki Okodo, Ayaka Shirasaki, Yurina Mikasa, Ryuta Iwamoto, Yuichi Takahashi, Fumiyoshi Kojima, Shin‐ichi Murata

**Affiliations:** ^1^ Department of Human Pathology Wakayama Medical University Wakayama Japan; ^2^ Department of Medical Technology Faculty of Health Sciences Kyorin University Tokyo Japan

**Keywords:** high‐grade, human papillomavirus, in situ hybridization, squamous differentiation, urothelial carcinoma

## Abstract

**Background:**

Human papillomavirus (HPV) is a well‐established mucosotropic carcinogen, but its impact on urothelial neoplasm is unclear. We aimed to clarify the clinical and pathological features of HPV‐related urothelial carcinoma (UC).

**Methods:**

Tissue samples of 228 cases of UC were obtained from the bladder, upper and lower urinary tract, and metastatic sites to construct a tissue microarray. The samples were analyzed for the presence of HPV by a highly sensitive and specific mRNA in situ hybridization (RISH) technique (RNAscope) with a probe that can detect 18 varieties of high‐risk HPV. We also conducted immunohistochemistry (IHC) for a major HPV capsid antibody and DNA‐PCR.

**Results:**

The HPV detection rates varied among the methods; probably due to low HPV copy numbers in UC tissues and the insufficient specificity and sensitivity of the IHC and PCR assays. The RISH method had the highest accuracy and identified HPV infection in 12 (5.2%) of the cases. The histopathological analysis of the HPV‐positive UC showed six cases of usual type UC, five cases of UC with squamous differentiation (UC_SqD), and one case of micropapillary UC. The HPV detection rate was six‐fold higher in the cases of UC_SqD than in the other variants of UC (odds ratio [OR] =8.9, *p* = 0.002). In addition, HPV infection showed a significant association with tumor grade (OR =9.8, *p* = 0.03) and stage (OR =4.7, *p* = 0.03) of UC. Moreover, the metastatic rate was higher in HPV‐positive than in negative UC (OR =3.4).

**Conclusion:**

These data indicate that although the incidence of HPV infection in UC is low, it is significantly associated with squamous differentiation and poor prognosis. Furthermore, our observations show that RNAscope is an ideal method for HPV detection in UC compared with the other standard approaches such as IHC and PCR assays.

## Graphical abstract

The etiological role of HPV infection in urothelial carcinoma is controversial. Moreover, its clinicopathological presentation and association with squamous differentiation are still undetermined. With highly specific and sensitive mRNA in situ hybridization assay, we detected HVP in a small subset of cases associated with poor prognosis and squamous differentiation. We also showed that methodologies variance was behind the discrepancy reported in previous studies.

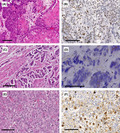

## 1 INTRODUCTION

The incidence of urinary bladder cancer has been increasing globally in recent decades.^1^ Urothelial carcinoma (UC), which accounts for about 90% of all bladder cancers, is by far the most common histological type worldwide. UC is characterized by spatiotemporal multiplicity, which may be reflected in differences in the urothelium as a result of carcinogen exposure or genetic factors. Several specific molecular pathways of carcinogenesis in UC were reported, such as alterations of the RTK/RAS/PI3K pathway, p53/Rb pathway, SWI/SNF complex, and histone modification.^2^, ^3^ Major risk factors for UC, such as cigarette smoking and environmental exposures have been identified. Recently, several studies have investigated the possible involvement of human papillomavirus (HPV) in UC.^4^, ^5^, ^6^ HPV is a well‐established mucosotropic carcinogen and a common cause of cancer in the anogenital region. It is implicated in about 90% of anogenital cancers.^7^ Although several case–control studies suggested the potential involvement of HPV in UC,^8^, ^9^, ^10^ the causality of HPV in UC was not established. Previous studies reported contrasting HPV detection rates of 0%–81% in UC.^10^, ^11^ The conflicting results are mainly due to the difference in methodological sensitivity and specificity and in the sample tissues.

The clinical and prognostic significance of HPV detection in UC remains unclear.^11^ Unlike in other HPV‐related cancers, the association of HPV with squamous differentiation of bladder tumor has not been shown. Studies on the detection of HPV in UC with squamous differentiation (UC_SqD) are very few, and evidence of this association is lacking. Most of the published studies detected only individual or no cases of HPV in UC_SqD.^12^, ^13^, ^14^, ^15^ Only one study detected an increase in HPV infection rates in cases of UC_SqD. However, the statistical significance of this association was not demonstrated.^16^ Moreover, the prevalence of HPV infection on the other histological variants of UC remains unknown.

Recently, a novel mRNA in situ hybridization (RISH) technique, the RNAscope®, has become commercially available. Compared with both immunohistochemical (IHC) and polymerase chain reaction (PCR) analyses, RISH is a robust method with the highest sensitivity (95%–98%) and specificity (93%–100%) in the detection of HPV infection.^17^, ^18^, ^19^ Furthermore, it enables the visualization of mRNA at the single‐cell level in formalin‐fixed and paraffin‐embedded (FFPE) sections under a light microscope. In this study, we applied the novel RNAscope technique to detect high‐risk (HR) HPV E6/E7 mRNA in paraffin‐embedded UC tissue sections. This study aimed to clarify the clinicopathological features of HPV‐related UC such as incidence, histological variants, histological grade, and prognosis in a large cohort involving 228 cases.

## 2 MATERIALS AND METHODS

### 2.1 Case selection and pathological review

We used our institutional pathology database to obtain the data of 228 FFPE tissue blocks from 217 patients with UC who consulted our institution between 2013 and 2019. Cases of pure squamous cell carcinoma, adenocarcinoma, or neuroendocrine tumors of the bladder were excluded. Tissue samples were obtained by means of transurethral resection of the bladder, cystectomy, or biopsy. Information on patient demographic, clinical, and pathological characteristics were collected. The selected cases were reassessed for their histological variant, grade, and stage classification in accordance with the 2016 WHO Classification.

### 2.2 Tissue microarray construction

First, we selected the most morphologically representative region of the tumor by examining hematoxylin–eosin‐stained slides. A tissue microarray (TMA) was constructed from the 228 FFPE tissue blocks according to the method described in our previous study.^20^ The TMA slides were stained with hematoxylin–eosin, while the unstained TMA slides were prepared for in situ hybridization and IHC.

### 2.3 RNA in situ hybridization (RISH, RNAscope)

The HR‐HPV E6/E7 mRNA was detected using the RNAscope® technology with HPV‐HR18 probe cocktail (Advanced Cell Diagnostics, Newark, CA, USA) that was used to recognize 18 HR (high‐risk) HVP genotypes, which included the following: 16, 18, 26, 31, 33, 35, 39, 45, 51, 52, 53, 56, 58, 59, 66, 68, 73, and 82. In the RNAscope® procedures, the RNAscope 2.5 HD‐BROWN manual assay kit (Advanced Cell Diagnostics) were performed according to the manufacturer's instructions. Briefly, 4‐mm thick TMA sections were air‐dried overnight, baked at 60℃ for 1 h, deparaffinized, and air‐dried again before pretreatment. The TMA sections were then boiled for 15 min in the target retrieval reagent and incubated for 30 min in RNAscope Protease Plus at 40℃ before hybridization. After pretreatments, the sections were hybridized with each probe in a HybEZ oven (Advanced Cell Diagnostics, Newark, CA, USA)) for 2 h at 40℃. The signal was visualized using the RNAscope 2.5 HD‐BROWN reagent kit. The *Homo sapiens* peptidylprolyl *cis*‐*trans* isomerase B and bacterial 4‐hydroxytetrahydrodipicolinate reductase (dapB) probes were used as positive and negative controls, respectively. Cervical squamous cell carcinoma tissue, which contained HR‐HPV, was also used as a positive control. In addition to the TMA sections, whole‐tissue sections of 40 selected cases, including the 12 positive TMA cases, were prepared and stained for RNAscope analysis using the same procedures. Slides of available preinvasive lesions from RISH‐positive cases were also evaluated for the presence of HPV infection with RNAscope®. Malignant cells with a higher intensity of punctate staining in the cytoplasm and/or nucleus compared with that of the negative control slide were regarded as positive. The RNAscope results were interpreted and classified using a 0 to 3+ scale by three of the authors (FYM, IM, and SM) of this manuscript following the manufacturer's instructions.

### 2.4 Immunohistochemistry (IHC)

HPV IHC detection was carried out on 5‐mm thick TMA sections using a Bond‐III automated IHC/ISH stainer (Leica Microsystems, Wetzlar, Germany). A primary polyclonal rabbit antibody against a major HPV capsid protein (clone K1H8, 1:100 dilution; Agilent Technologies, Palo Alto, CA, USA) was used to detect the following 11 HPV genotypes: 6, 11, 16, 18, 31, 33, 42, 51, 52, 56, and 58. HPV‐positive cervical carcinoma sections were used as positive controls. The presence of a cytoplasmic staining was regarded as positive for HPV. The detection intensity was interpreted and classified as 0 to 3+ by three of the authors (FYM, IM, and SM) of this manuscript.

### 2.5 Uniplex E6/E7 polymerase chain reaction (PCR) analysis

HPV genotyping was conducted by means of PCR analysis, which was performed on 55 samples, including 32 HPV‐positive and 23 randomly selected HPV‐negative samples that were confirmed by the results of either the RNAscope® technique or IHC. In one case (case 5), two FFPE samples of the same tumor taken at different stages and times were simultaneously analyzed.

DNA was extracted from FFPE tissue sections of the corresponding cases and analyzed with uniplex E6/E7 PCR method following a previously described procedure.^21^ HPV type‐specific primers for the E6 and E7 genes were designed for 39 common HPV types, including HPV‐61, −62, −81, −84, and −89 (Alpha‐3); HPV‐26, −51, −69, and −82 (Alpha‐5); HPV‐30, −53, −56, and −66 (Alpha‐6); HPV‐18, −39, −45, −59, −68, −70, and −85 (Alpha‐7); HPV‐16, −31, −33, −35, −52, −58, and −67 (Alpha‐9); HPV‐6, −11, −44, −55, and −74 (Alpha‐10); HPV‐34 and −73 (Alpha‐11); HPV‐42 and −54 (Alpha‐13); and HPV‐71 and HPV‐90 (Alpha‐15). The PCR mixture included 1×Ex Taq buffer (Mg2+ plus), 0.2 mmol/L dNTP, 0.03 U/μL TaKaRa Ex Taq HS (Takara Bio, Shiga, Japan), 2.5 μL DNA, and 0.5 pmol/L primers in a total volume of 25 μL. PCR amplification was performed using a thermal cycler with the following settings: 40 cycles of denaturation at 95℃ (30 s) annealing at 60℃ (30 s), and extension at 72℃ (30 s), initial denaturation step (5 min) and a final extension step for 5 min. Human β‐actin expression, which was simultaneously determined using a different PCR method, was used as the internal standard. A total of 4 μL of each reaction solution was applied on a 2% agarose gel (Bio‐Rad, Hercules City, CA, USA) and was electrophoresed in 1× Tris/borate/EDTA buffer. The HPV type‐specific bands were visualized using the SYBR® Green I staining (Takara Bio Shiga, Japan) and observed under UV light.

### 2.6 Statistical analysis

The association between HPV infection and clinicopathological parameters was statistically assessed using the Fisher's exact test. The sample *t*‐test was used for the comparison of means. Results were considered statistically significant if the *p* value was <0.05. The JMP Start Statistics version 13 (Statistical Discovery Software; SAS Institute, Cary, NC, USA) was used for statistical analyses.

## 3 RESULTS

### 3.1 Clinicopathological characteristics of the cohort study

The cohort study (N = 228 cases) was composed of 182 (79.8%) male and 46 (20.2%) female patients with a mean age of 74.8 years (Table 1). Based on the WHO classification, the included histological variants were UC of usual type (184 cases), UC with squamous differentiation (UC_SqD,19 cases), UC with glandular differentiation (3 cases), UC with divergent squamous and glandular differentiation (2 cases), micropapillary UC (9 cases), sarcomatoid UC (6 cases), nested UC (2 cases), lymphoepithelioma‐like UC (2 cases), and UC with trophoblastic differentiation (1 case) (Table 2). Low‐ and high‐grade UC were found in 61 (26.8%) and 167 (73.2%) cases, respectively. Noninvasive UC was the most frequent tumor (39.5%, including 37.3% pTa and 2.2% pTis), followed by invasive pT1 (21.9%), pT2 (18.0%), pT3 (11.4%), pT4 (3.1%), and metastatic (6.1%) tumors.

**TABLE 1:** Clinical characteristics of the study cohort

Items	N (%)
Total case	228
Age, mean (SD)	74.8 (9.1)
Sex	
Male	182 (79.8)
Female	46 (20.2)
Tumor site	
Primary site	162 (71.1)
Bladder	
Upper urinary tract	42 (18.4)
Urethra	6 (2.6)
Invasive and Metastatic site	
Perivesicular extension	5 (2.2)
Lung	7 (3.1)
Liver	3 (1.3)
Colon	1 (0.4)
Soft tissue	1 (0.4)
Testis	1 (0.4)
Bone	1 (0.4)

**TABLE 2:** Pathological characteristics of the study cohort

Items	N (%)
pT stage	
pTa	85 (37.3)
pTis	5 (2.2)
pT1	50 (21.9)
pT2	41 (18.0)
pT3	26 (11.4)
pT4	7 (3.1)
pTxM1	14 (6.1)
Histological grade	
High	167 (73.2)
Low	61 (26.8)
Histological variant	
Usual type	184 (80.7)
Squamous differentiation	19 (8.3)
Divergent squamous and glandular differentiation	2 (0.9)
Micropapillary	9 (3.9)
Sarcomatoid	6 (2.6)
Glandular differentiation	3 (1.3)
Nested	2 (0.9)
Lymphoepithelioma‐like	2 (0.9)
Trophoblastic differentiation	1 (0.4)

### 3.2 Comparison of IHC, PCR, and RISH results

**FIGURE 1:**
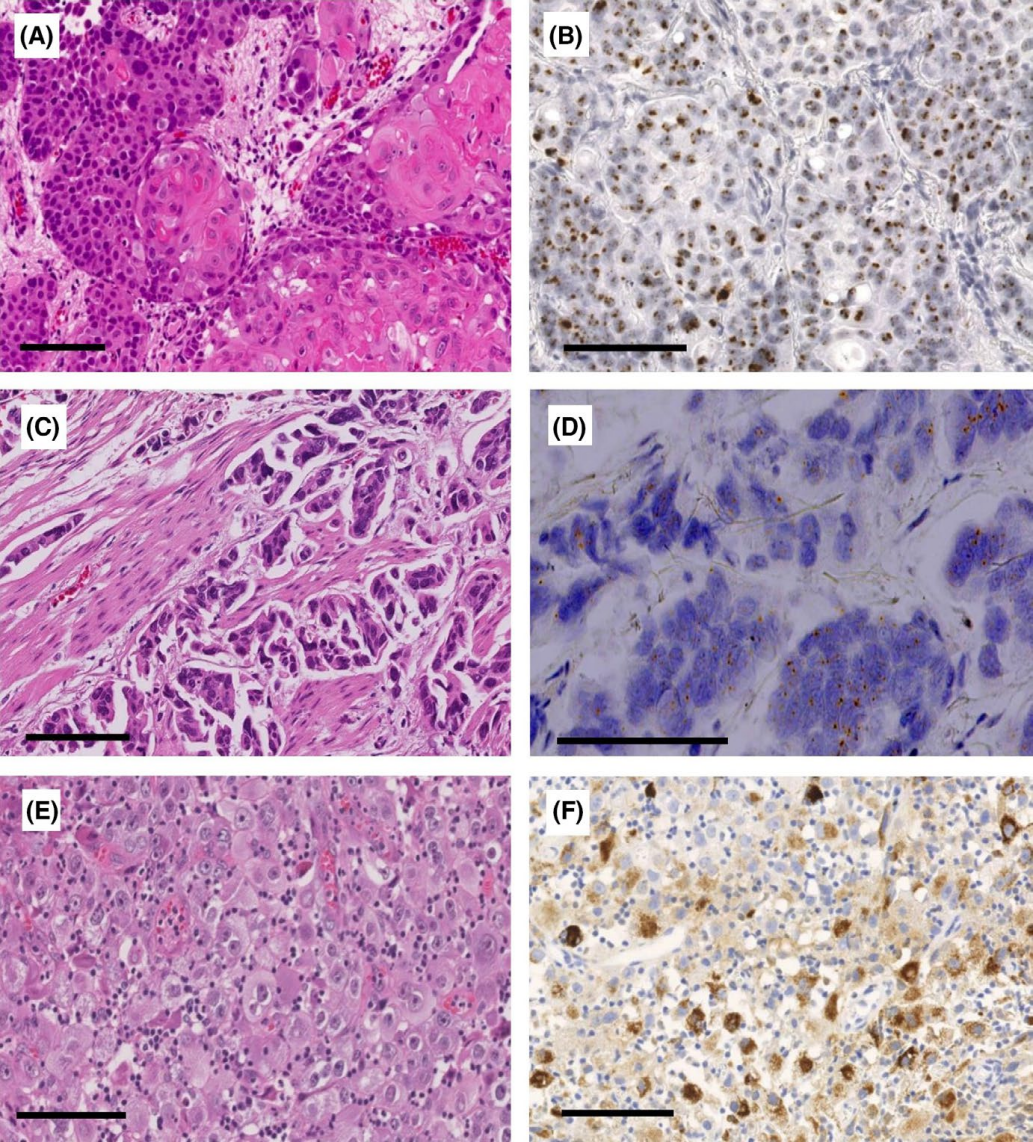
Representative light microscopy images of HPV‐associated urothelial carcinoma (UC). An invasive UC with squamous differentiation (HE stain, A), showing nuclear positivity for high‐risk HPV E6/E7 mRNA by ACD RNAscope® Probe HPV HR18. (RISH stain, B) Muscle‐invasive micropapillary UC (HE stain, C), showing positive brownish punctate staining in the cell nuclei and cytoplasm for high‐risk HPV E6/E7 mRNA by RNAscope (RISH stain, D). An invasive high‐grade UC (HE stain, E), showing strong cytoplasmic immunohistochemical reaction for a major HPV capsid antigen (IHC stain, F). Scale bars represent 100 μ m

**FIGURE 2:**
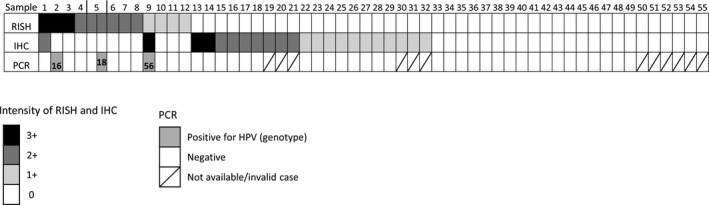
Comparison of human papillomavirus (HPV) analyses by mRNA in situ hybridization (RISH), Immunohistochemistry (IHC), and polymerase chain reaction (PCR) in 55 cases of urothelial carcinoma. The HPV genotypes (16, 56, and 18) of the three samples testing HPV‐positive by PCR are indicated. Additional 173 negative cases analyzed only by RISH and IHC are not shown here

RISH with HPV‐HR18 probe cocktail detected 12/228 (5.2%) HPV‐positive cases. In the positive tissue samples, RISH examination revealed various numbers of brownish dots in the cytoplasm and nucleus of the majority of tumor cells (Fig. 1B, D). IHC with polyclonal antibody for HPV capsid antigen showed positive staining in 22/228 (9.6%) cases. IHC analysis revealed weak to strong cytoplasmic reaction in positive tissue samples (Figure 1E). However, due to its low specificity, IHC staining for the HPV capsid antigen also showed considerable background staining on benign tissue components, thereby rendering it more likely to yield false‐positive results. Indeed, 20/22 of IHC‐positive cases were deemed HPV negative using both RISH and PCR. The PCR test detected HPV DNA in only 3/55 cases (genotypes 16, 18, and 56; Figure 2).

The three PCR‐positive cases were RISH‐positive but only one (genotype 56) was also IHC‐positive. In one patient (case 5, genotype 18), PCR assay showed a variation of results of the same tumor (PCR‐negative in the early pT2 tumor sample and PCR‐positive in the late pT3 sample) while RISH showed a constantly positive tumor. The PCR assay did not detect HPV DNA in any of the RISH‐negative cases. RISH assay detected HPV in 9/52 (17%) of PCR‐negative/invalid cases.

### 3.3 Clinical features of HPV RISH‐positive cases

The clinical characteristics of the 12 HPV RISH‐positive patients are summarized in Table 3. The mean age was 76.8 years with a male to female ratio of 3:1. Among the 12 HPV‐positive cases, 10 were primary tumors. A total of nine cases were taken from the urinary bladder, while one case was from the renal pelvis. Smoking and diabetes mellitus were reported in 58% (7/12) and 50% (6/12) of the cases, respectively. An underlying bladder condition was found in one patient who presented with multiple bladder diverticula and UC with divergent squamous and glandular differentiation. No underlying anatomical (defects, chronic trauma, or catheterization) or functional (neurogenic bladder) bladder condition were reported on the other 11 cases. In two patients no medical history of diabetes mellitus, smoking, or underlying bladder condition was found.

**TABLE 3:** Clinicopathological characteristic of RISH HPV‐positive cases

Case No.	Age	Sex	Stage	Site	Histological variant	Associated preinvasive lesion HPV status	Smoking	DM	Underlying bladder condition
1	77	M	pT3	Bladder	Divergent Squamous and glandular differentiation	CIS +	Yes	Yes	Bladder diverticula
2	73	F	M1	Lung	Usual type	NA	No	Yes	No
3	70	M	pT3	Bladder	UC_SqD	CIS +	No	Yes	No
4	76	M	pT1	Bladder	UC_SqD	HG pTa_SqD +	Yes	No	No
5	67	M	pT3	Bladder	Usual type	CIS +	Yes	Yes	No
6	75	M	pT3	Bladder	UC_SqD	AUS ‐	No	Yes	No
7	87	F	pT2	Bladder	UC_SqD	NA	No	No	No
8	86	F	pT2	Bladder	Usual type	NA	Yes	No	No
9	90	M	pT2	Bladder	Micropapillary	NA	Yes	No	No
10	88	M	pT1	Bladder	Usual type	CIS +	Yes	No	No
11	77	M	pTis	Renal pelvis	Usual type	CIS +	Yes	Yes	No
12	55	M	M1	Testis	Usual type	NA	No	No	No

AUS indicates Atypia of unknown significance; CIS, carcinoma in situ; DM, diabetes mellitus; HG pTa_SqD, high grade noninvasive papillary urothelial carcinoma with squamous differentiation; HPV, human papillomavirus; NA, not available; RISH, mRNA in situ hybridization; UC_SqD, urothelial carcinoma with squamous differentiation; +, positive; ‐, negative.

### 3.4 Pathological features of the HPV‐positive RISH cases

The 12 HPV‐positive cases, which were confirmed by RISH, were high‐grade tumors. Among these, 75% (9/12) were muscle‐invasive or metastatic (Table 3). Histopathological analysis revealed six cases of UC of usual type, four cases of UC_SqD (Figure 1A, B), one case of UC with divergent squamous and glandular differentiation, and one case of micropapillary variant (Figure 1C, D).

Squamous differentiation was found in almost half (5/12) of the positive cases. The areas of squamous differentiation were predominant (>50%) in one case and focal (<50%) in the other four.

Of the 12 HPV RISH‐positive cases, associated preinvasive urothelial lesions were histologically available in seven cases. In RISH, HPV was detected in 6/7 (85%) of associated preinvasive lesions. The six HPV ‐positive preinvasive lesions included five (83%) flat high‐grade lesions consistent with urothelial carcinoma in situ (UCIS) and one high‐grade noninvasive papillary urothelial carcinoma with squamous differentiation. The only negative associated preinvasive lesion was morphologically consistent with a flat lesion with atypia of unknown significance.

### 3.5 Correlations between HR‐HPV and the clinicopathological parameters of UC patients

Clinicopathological comparisons between HPV‐positive and HPV‐negative cases using the RNAscope® revealed no significant difference in terms of age (76.8 ± 10.1 vs. 74.7 ± 9.1, *p* = 0.49) and male to female ratio (odds ratio [OR] =0.74, *p* = 0.71) (Table 4). No significant difference was found in the two groups in terms of the tumor site. In addition, there were no HPV‐positive tumors in samples obtained from the urethra (0/6).

**TABLE 4:** Clinicopathological comparison of cases with respect to HPV detection by RISH

		RISH Negative (%)	RISH positive (%)	p value
Total cases		216	12	
Age, mean (SD)		74.7 (9.1)	76.8 (10.1)	0.49
Sex				0.71
Male		173 (80.1)	9 (75)	
Female		43 (19.9)	3 (25)	
pT stage				
Non‐muscle invasive tumor		137 (63.4)	3 (25)	0.03
Muscle invasive tumor		67 (31.0)	7 (58.3)	0.03
Metastatic tumor		12 (5.6)	2 (16.7)	0.16
Grade				0.03
Low‐grade		61 (28.2)	0	
High‐grade		155 (71.8)	12 (100.0)	
Variant				
Usual type		178 (82.4)	6 (50.0)	0.01
Squamous differentiation / features^a^		16 (7.4)	5 (41.7)	0.002
Glandular differentiation		3 (1.4)	0	
Micropapillary		8 (3.7)	1 (8.3)	0.39
Sarcomatoid		6 (2.8)	0	
Others		5 (2.3)	0	
Tumor site				
Primary site	Urinary bladder	153 (70.8)	9 (75.0)	1
	Upper urinary tract	41 (19.0)	1 (8.3)	0.7
	Urethra	6 (2.8)	0	
Invasive and metastatic site	Perivesicular extension	5 (2.3)	0	
	Lung	6 (2.8)	1 (8.3)	
	Testis	0	1 (8.3)	
	Others	1 (0.5)	0	

Abbreviations, HPV: Human papilloma virus, RISH: mRNA in situ hybridization.

^a^ Variant with squamous differentiation / features includes urothelial carcinoma with squamous differentiation and urothelial carcinoma with divergent squamous and glandular differentiation.

The proportion of UC with squamous features (squamous differentiation and divergent squamous and glandular differentiation) was significantly higher in HPV‐related UC than in HPV‐negative cases (OR =8.9, *p* = 0.002). The HPV detection rate was sixfold higher in cases of UC_SqD than in usual and other histological subtypes of UC (23.8% vs. 3.5%).

**FIGURE 3:**
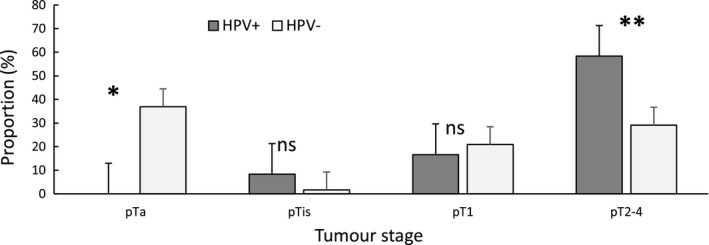
Percent distribution of tumor stages among human papillomavirus (HPV)‐negative (N=216) and HPV‐positive groups (N=12). Error bars represent the standard error. ns: non‐significant, ^*^ *p* = 0.004, ^**^ *p* = 0.03

Compared with HPV‐negative cases (71.8%), all HPV‐positive cases (100%) showed a high histological grade (OR =9.8, *p* = 0.03). Furthermore, the proportion of muscle‐invasive tumors (pT2 to pT4) was significantly higher in HPV‐positive than in HPV‐negative cases (OR =4.7, *p* = 0.03, Figure 3). The metastatic rate was higher in HPV‐positive UC patients (16.7% vs. 5.6%), but the difference was not significant (OR =3.4, *p* = 0.16).

## 4 DISCUSSION

HPV an established carcinogen of squamous cell carcinoma in certain anatomical mucosa such as the uterine cervix, anogenital skin, and oropharyngeal mucosa. On the other hand, several studies reported the potential involvement of HPV infection in squamous cell carcinoma in other anatomical sites.^20^, ^22^ The majority of published studies on UC suggested a relationship between HPV and UC carcinogenesis in subset of patients.^11^ However, there is still discordance in the incidence of HPV‐related UC and their clinicopathological characteristics, including histological subtype, tumor grade, and stage.

Numerous methods have been developed for detecting HPV in biological samples. Among them, IHC, PCR, and in situ hybridization methods are the most common assays. There are many different ISH methodologies, which use different types of probes, DNA or RNA targets, and visualizing procedures. The recently developed mRNA ISH method, the RNAscope®, offers the highest specificity and sensitivity for the detection of HPV infection compared to other methods tested, and it is currently considered the gold standard among HPV detection assays.^17^, ^18^, ^19^, ^23^, ^24^ The RNAscope® technique has additional advantages in providing information on the associated morphology and on the detection of integrated and transcriptionally active HPV in each cell.

Differences in sensitivity and specificity among the various methods have resulted in significant differences in HPV detection rates among RISH, DNA‐ISH, PCR, and IHC assays in previous studies.^17^ In addition, in the current study, the IHC assay was conducted using an HPV major capsid polyclonal antibody, which detected 22 (9.6%) positive cases, but only two of the 22 cases were simultaneously positive in the RISH assay. However, a considerable nonspecific background staining was also noted on benign tissue component. Similar findings have been reported by Lopez‐Beltran et al.^25^ who analyzed HPV in 76 cases of UC and found a positivity rate of 32.8% using the IHC for the HPV capsid antigen using a polyclonal antibody, whereas only 15.7% of the cases were positive for HPV 16/18‐DNA using non‐isotopic in situ hybridization. These findings are more likely due to the nonspecific binding of the IHC HPV capsid antibody and its lower sensitivity in advanced HPV‐related neoplasm with integrated viral DNA.^14^, ^26^ In fact, a number of HPV IHC tests with antibodies against either E6 or E7 oncoproteins or HPV L1 capsid protein have not yet been validated for clinical use in HPV detection.

On PCR assay, HPV DNA was detected in only three cases, all of which were HPV RISH‐positive. In one of the patients, the PCR yielded variations in the results of the two FFPE tissue samples from the same tumor of different stages, whereas the RISH results were constantly positive. For this patient, the PCR‐positive result came from a tissue sample with a higher tumor stage and the most recent FFPE block. Aside from that, RISH detected HR‐HPV in 17% of PCR‐negative/invalid cases. This discordance is very likely due to higher false negative results in the PCR assay, which is more likely to be affected by preanalytical factors such as increasing block age,^17^, ^27^ especially in malignancies with low viral copy numbers. This is in line with the previous findings by Mills et al. who compared the performance of the HPV RISH assay to DNA‐PCR, p16 IHC, and DNA‐ISH assays in HPV‐related malignancies and found a higher sensitivity and specificity for RISH, which detected HR‐HPV infection in 18% of PCR‐negative cases and 38% of PCR invalid cases.^18^ Several studies reported that the PCR assay had a lower HPV detection compared with RISH in non‐anogenital cancers, including bladder neoplasm with a low HPV DNA copy number.^19^, ^28^, ^29^ The findings in our study and in previous reports suggest that RISH, which is more specific and sensitive than standard IHC and DNA‐PCR assays, is a reliable method for detecting HPV in FFPE tissue from non‐anogenital or oropharyngeal malignancies with low HPV copy number.^19^, ^28^ Based on the above considerations, we used only the results of the RISH assay for definite clinicopathological assessment of HPV cases in the current study. On RISH analysis, HR‐HPV was detected in 12 different cases and in 85% of their associated preinvasive lesion.

HPV carcinogenesis results from the combination of local (chronic inflammation, trauma) and systemic (immune response, smoking) host factors that promote the persistence, integration, and replication of the viral material. To assess the presence of potentially associated risk factors of HPV infection in UC, patients were screened for the presence of underlying bladder condition, smoking history, or diabetes mellitus.

Interestingly, one patient had multiple bladder diverticula and UC with divergent squamous and glandular differentiation. In the literature, bladder neoplasm has been identified in 0.8% to 10% of patients with bladder diverticula.^30^ However, to the best of our knowledge, this is the first study that described HPV‐driven UC in this setting. In addition, we also found a high frequency (50%) of diabetes mellitus in HPV‐positive cases. Previous reports on HPV‐related UC did not explore its possible relationship with diabetes mellitus. Nevertheless, a similar finding was observed in HPV‐related neoplasm on other anatomical sites.^31^, ^32^ Sobti et al.^32^ found that HPV‐16‐positive head and neck squamous cell carcinoma were significantly associated with a higher prevalence (45%) of diabetes mellitus compared with HPV‐negative (27%) cases. Diabetes mellitus causes an immunosuppressive condition linked to an impaired innate and acquired immunity, which when coupled with glucosuria may promote bladder inflammation and HPV carcinogenesis. Further studies are however needed to clarify this relationship.

Analysis of HPV RISH‐positive patients showed the same demographic characteristics as HPV‐negative patients in terms of age and sex in our study. A previous study reported differences in HPV detection with age.^33^ The majority of previous investigations have reported a male predominance and the same age range in HPV‐related UC.^4^ These results are in line with the reported evidence that male sex and age are major unmodifiable risk factors for UC, independent of the exposure‐related risk factors of HPV.

Almost half of the positive cases in our study had squamous differentiation. The HPV detection rate was six‐fold higher in UC_SqD cases compared with the usual and other histological variant of UC. To our knowledge, this study is the first to show a significant association between HPV infection and squamous differentiation in UC. Among the known special variants of UC, UC_SqD is the most common type reported in up to 21% of invasive urothelial carcinoma.^34^ In our study, HR‐HPV was detected in 23% of UC_SqD and their corresponding precursor lesions. Data on the association between HPV infection and squamous differentiation in UC are very limited and contradictory in previous reports. Using both IHC and DNA‐ISH methods in the urinary bladder, Alexander et al. found no HPV infection involvement in 27 UC_SqD cases and 42 squamous cell carcinoma cases.^15^ Conversely, Kim et al. found a two‐fold higher detection rate of HPV in a group of UC_SqD patients than in the control group using a highly sensitive HPV DNA chip.^16^ The conflicting results on this subject may be more likely attributable to methodological and sampling issues than to the geographical variation in HPV prevalence. Besides, among other histological variants, we surprisingly found an HPV‐positive UC case of micropapillary variant, which is also known to harbor frequent ERBB2 amplification and is associated with poor outcome.^35^ This is the first report of an HPV‐related UC case of micropapillary variant. Although this single positive case was not sufficient to assume an association between the variant and HPV infection, this finding may suggest that some of the HPV‐related urothelial tumors could follow similar pathways of urothelial tumorigenesis.

The most significant prognostic factors of UC include tumor grade, stage, and the presence of CIS.^36^ Interestingly, all 12 HPV‐positive tumors in our current study were high‐grade. A similar trend has been found in several previous studies.^37^, ^38^, ^39^ Golovina et al. reported that 97.4% of HPV‐positive cases identified by PCR assay were high‐grade tumors.^39^ Moreover, HPV infection was significantly correlated with high tumor stage in the present study. We found a significantly higher rate of muscle‐invasive tumors in HPV‐related cases than in non‐HPV‐related cases. We also found a higher proportion of metastatic cases in HPV‐positive cases compared with that in HPV‐negative cases, even though statistical significance was not established because of the small number of metastatic cases. While some studies found no association between tumor stage/grade and HPV‐related UC,^28^, ^40^ others have revealed a correlation between higher tumor grades and stages and HPV infection.^5^, ^37^, ^38^ In a study carried out by Lopez‐Beltran, the majority of HPV‐positive UC patients (71.4%) presented with high pathological stage and grade and poor survival.^37^ In addition, Moonen et al. detected HR‐HPV infection rates of 0%, 12.5%, and 18.2% for Ta, T1, and T2–T4 tumors, respectively, by PCR assay on frozen bladder samples, and found a significant correlation between grade/stage and HR‐HPV infection.^38^

Further, in the current study, 5/6 (83%) of HPV‐positive associated preinvasive lesions consisted of UCIS which are known to be associated with an increased risk of tumor recurrence and progression.^36^ Taken together, these results showed that HPV‐related UC demonstrated an aggressive phenotype with higher grade and stage and poor prognosis.

In conclusion, there were contradictory data in past reports regarding the incidence of HPV infection in UC and the association between HPV infection and clinicopathological factors. This may be due to differences in sensitivity and specificity of the detection method used. To detect low HPV copy numbers in non‐anogenital or oropharyngeal malignancies such as UC, highly specific and sensitive methods must be used, such as RNAscope®, which we used in the present study. Based on our data, HPV infection is detected in only a subset UC in association with squamous differentiation and higher histological grade and tumor stage. Our study provided new insights into the clinical and pathological presentation of HPV‐related UC and their potentially associated risk factor. However, larger case‐control and multicenter studies are needed to clarify the etiological role and carcinogenesis of HPV infection in UC.

## ETHICAL APPROVAL

Institutional review broad approval was obtained at Wakayama Medical University Hospital, and informed consent was obtained in the form of opt‐out on the website.

## CONFLICT OF INTERESTS

The authors declare no conflict of interests.

## AUTHOR CONTRIBUTIONS

FYM, IM, and SM conceived and designed the study. IM, MO, and AS collected, organized, and reported data and performed the laboratory work. MO did PCR analysis. FYM, IM, and SM analyzed and interpreted the data. FYM, IM, MO, YM, RI, YT, FK, and SM wrote or reviewed the manuscript. SM supervised the study. All authors read and approved the final manuscript.

## Data Availability

The data that support the findings of this study are available from the corresponding author (S.M), upon reasonable request.
